# Infections of Dwarf Tapeworm *Hymenolepis nana* (Cestoda: Hymenolepididae) and Its Associated Risk Factors Among Internally Displaced Children in Deynile District, Mogadishu, Somalia

**DOI:** 10.1155/japr/1526083

**Published:** 2026-04-27

**Authors:** Mohamed Adam Mahamud, Nasteho Ali Isse, Abdirizaq Adan Ali, Abdikani Omar Salah, Ahmed Mahad Sheikh Mohamed, Jamal Hassan Mohamoud, Mohamed Hussein Adam, Bashiru Garba, Hodo Aideed Asowe, Arwa Elaagip

**Affiliations:** ^1^ Department of Microbiology and Laboratory Sciences, Faculty of Medicine and Health Sciences, SIMAD University, Mogadishu, Somalia, upm.edu.my; ^2^ Department of Public Health, Faculty of Medicine and Health Sciences, SIMAD University, Mogadishu, Somalia, upm.edu.my; ^3^ SIMAD Institute for Global Health, SIMAD University, Mogadishu, Somalia, simad.edu.so; ^4^ Department of Nursing and Midwifery Science, Faculty of Medicine and Health Sciences, SIMAD University, Mogadishu, Somalia, upm.edu.my; ^5^ Department of Parasitology and Medical Entomology, Faculty of Medical Laboratory Sciences, University of Khartoum, Khartoum, Sudan, uofk.edu

**Keywords:** children, *Hymenolepis nana*, internally displaced persons (IDPs), prevalence, risk factors, Somalia

## Abstract

**Background:**

Hymenolepis nana remains a major public health problem in areas with inadequate sanitation and limited access to healthcare. The infection is common in developing countries with high fecal–oral transmission rates. In Somalia, frequent internal displacement caused by conflict and natural disasters increases the vulnerability of children living in internally displaced person (IDP) camps.

**Objective:**

This study is aimed at determining the prevalence of *H. nana* infection and identifying associated risk factors among displaced children residing in IDP camps in the Deynile district of Mogadishu, Somalia.

**Methods:**

A community‐based cross‐sectional study was conducted from February to March 2024 among children aged 6 months–15 years in two IDP camps: Iga‐Daba Geey and Jilyaal. A total of 384 children were selected using a convenient sampling method. Data were collected through structured questionnaires covering sociodemographic information, hygiene practices, and potential risk factors. Stool samples were examined microscopically for *H. nana* eggs using the saline wet mount technique. Logistic regression analyses were performed to identify statistically significant risk factors.

**Results:**

The overall prevalence of *H. nana* infection was 27.08%, with males being significantly more affected than females (*p* = 0.003). Children from Iga‐Daba Geey had higher infection rates compared with those from Jilyaal (*p* = 0.004). Significant risk factors included large family size (> 5 members) (AOR = 6.96, *p* < 0.0001), playing on unclean grounds (AOR = 3.92, *p* = 0.030), fingernail biting (AOR = 3.25, *p* = 0.002), and consumption of unwashed vegetables and fruits (AOR = 8.16, *p* < 0.0001).

**Conclusions:**

This study reveals a high prevalence of *H. nana* infection among children in IDP camps in Mogadishu. The findings underscore the need for targeted public health interventions, including improved sanitation, hygiene education, and regular deworming programs, to reduce infection risks among displaced populations.

## 1. Background

Soil‐transmitted helminth infections remain highly prevalent worldwide, with an estimated 1.5 billion infections, particularly in impoverished and underserved communities [1]. Hymenolepiasis, caused by the dwarf tapeworm *Hymenolepis nana*, is a neglected zoonotic disease significantly contributing to the global burden of intestinal parasitic morbidity, particularly in low‐income settings with poor sanitation [2, 3]. Its incidence is predominantly high in developing countries with poor sanitary conditions, including usage of contaminated drinking water, improper sewage disposal, and poor personal hygiene [4,5]. *H*. *nana* infection disproportionately affects young children [6], and has a considerable impact on normal development, well‐being, as well as cognitive and educational performance of school age children [6,7]. These children may experience prolonged diarrheal episodes (dysentery), and may even lead to death [8]. Furthermore, chronic hymenolepiasis infections have become the subject of speculation and investigation in relation to the spreading and severity of other infectious diseases such as HIV/AIDS and leprosy [9, 10].

The dwarf tapeworm has a small size, 2–4 cm long, and only 1 mm wide. The adult tapeworm parasitizes the small intestine of humans and releases eggs excreted in the stool [10]. Mono‐infections are rare and usually *H. nana* is reported as co‐occurring with a variety of other enteric parasites, particularly in community situations where the frequency of fecal–oral transmission is likely to be high [9].

*H. nana* has a unique life cycle that may be direct or indirect. In the direct cycle, infection follows ingestion of embryonated eggs, with larval development (cysticercoid stage) occurring within the intestinal villi of the same human host, allowing humans to function as both definitive and intermediate hosts [4] In the indirect cycle, arthropods serve as intermediate hosts, and transmission occurs when infected arthropods containing cysticercoid larvae are ingested [11, 12]. The infection can have an important epidemiological impact in family units because of auto‐reinfection that occurs particularly in environments where the frequency of such transmission is likely to be high [9]. Another important factor that affects its prevalence is the presence of asymptomatic people in the community who can be considered as the main source of infection through continuously excreting the eggs with their stools [6].

There is broad diversity of clinical symptoms in patients with hymenolepiasis. Mild infections of *H. nana* are asymptomatic but severe infections cause abdominal pain, vomiting, emaciation, headache, diarrhea, dizziness, anorexia, loss of appetite, itching around the anus, irritability, and behavioral disturbances [10, 13]. Allergic reactions, such as urticarial, skin eruption, and phlyctenular conjunctivitis, may also occur [12].

The associated risk factors can be limited access to clean water, poor sanitation, open defecation, poor personal hygiene, overcrowding, low household income, knowledge on transmission, eating raw vegetables, and encountering contaminated soils [10].

Diagnosis is based on the demonstration of the characteristic eggs in the feces by direct smear [12]; the eggs can readily be concentrated by the salt flotation and formalin–ether sedimentation methods [14]. Some drugs are available for the treatment of *H. nana* infection, including praziquantel [15], nitazoxanide, and niclosamide [16]. Little can be done to control the disease in the endemic areas of high transmission, which requires case diagnosis and management, education, improved hygiene and sanitation [10].

As a result of insecurity, armed conflict, war, violence, violations of human rights or natural disasters in many African regions, many people from the affected areas were displaced to live in IDP camps in the more safe and secure regions [17]. Internal displacement in sub‐Saharan Africa was higher in recent years, with children being a particularly vulnerable group of IDPs because of their unique health needs. Therefore, they may be left unsupported by general health service provision [18].

Many countries such as Afghanistan, Iraq, Libya, Somalia, Sudan, Syrian Arab Republic, and Yemen have experienced massive crisis, continuous movements, forced migration, and forced displacement, and contributed to the highest rates of internal displacement worldwide. These high rates of mobility have increased the need for access and utilization of health care services [19].

Somalia with a total population of 18.7 million people experienced the worst drought and most extensive floods in decades in 2023. Despite some improvements, levels of humanitarian needs are still severe and extreme. Almost one in five Somalis face high levels of acute food insecurity [20].The poor socioeconomic conditions in the IDPs camps have undoubtedly resulted in high prevalence of at least some of the soil transmitted helminths [14].

Epidemiological surveys to determine the prevalence of *H. nana* infections and associated risk factors are of high importance, especially those targeting vulnerable communities. *H*. *nana* affects growth, wellness, and development of children; thus, more studies are needed to provide evidence‐based data to health authorities to assist in the design of appropriate strategies in controlling *H. nana* infections in the study areas. The aims of the present study were to determine the prevalence of *H. nana* infections among children in IDPs camp and to identify the associated risk factors in Deynile district, Mogadishu, Somalia.

## 2. Methods

### 2.1. Study Design

A community‐based cross‐sectional study was conducted from February to March 2024 to determine the prevalence of *H. nana* infections and their associated risk factors among children of displacement communities in Deynile district, Mogadishu, Somalia.

### 2.2. Study Area

Deynile district is one of the most well‐known in Benadir Region and the second largest districts in the Mogadishu, Somalia. It is located to the west of Mogadishu and covering an estimated area of 67 km^2^. It was officially recognized as a district in 1974 and consists of six localities, including Garasbaley, Kurdamac, Ciise Cabdi, Barwaaqo, Dibileey, and Halgan (Ex‐Raadaarka). The district houses are occupied majority by internally displaced people in Mogadishu [21]. The district has more than 1300 verified IDP sites with over 159,525 households and approximately 739,137 internally displaced people. Most of these IDP camps are privately run, spontaneous settlements that are often overcrowded, and lack basic sanitation and health services [22]. Two IDP camps were selected for the present study: Iga‐Daba Geey and Jilyaal in Deynile district, Mogadishu, Somalia, because of previous observations of high infections and overcrowding of the two camps.

### 2.3. Study Participants

The study population consisted of all children aged 6 months–15 years who lived in the Iga Daba‐Geey and Jilyaal camps and met the inclusion criteria. The inclusion criteria included children aged between 6 months and 15 years who had lived in the aforementioned two camps for at least 6 months and agreed to participate voluntary to the study after signed the consent form. We excluded any participants who did not consent to the study or who had taken antiparasitic medications within the 3 months preceding the sampling.

### 2.4. Sampling Technique and Study Sample Size Determination

The study employed a community‐based cross‐sectional design using a multistage sampling approach. Two IDP camps were purposively selected due to overcrowding and increased risk of infection. Within these camps, households were selected using systematic random sampling from camp records. In each selected household, one eligible child was included; where multiple children were present, one was selected using a simple random method. In the absence of prior prevalence data for *H*. *nana*, the sample size was calculated using a single population proportion formula (*P* = 50*%*, 95% CI, *d* = 5*%*), yielding a minimum sample of 384.

### 2.5. Questionnaire Survey

A structured questionnaire was developed to collect relevant information from each participant. Two domains were addressed in the questionnaire: sociodemographic (such as age, sex, location, family size, and education level) and risk factors for *H. nana* infections (including the source of drinking water, contact with soil, fingernail‐biting habits, type of toilet, handwashing before and after meals, and consumption of fresh vegetables).

### 2.6. Stool Sample Collection and Laboratory Procedures

Stool samples were collected from children in households selected. Before data collection, meetings were conducted at the selected IDPs camps to inform residents, family heads, and potential participants about the study′s objectives and methods. The day before sample collection, parents of consenting children received clean, prelabelled, capped plastic containers and instructions for stool collection, which was to occur between 8 and 11 a.m. Each sample was checked for correct labelling and adequate quantity, and then transported to Laboratory of Parasitology at SIMAD University within 15 min using tuk‐tuks. In the laboratory, although more sensitive techniques such as Kato–Katz and stool concentration methods are recommended for helminth diagnosis, Kato–Katz was not available in the study setting and stool concentration methods were not feasible due to local laboratory constraints; therefore, saline wet mount microscopy was used as the routine diagnostic method available in public health laboratories in Somalia. For the direct saline wet mount, two drops of 0.85% normal saline were placed on a clean glass slide, and a small amount of fresh stool was added using a wooden applicator stick. The specimen was mixed gently, covered immediately with a coverslip to prevent drying, and examined microscopically. Two wet mount slides were prepared from each stool sample and examined independently by two trained senior technologists using 10× and 40× objective lenses for the detection of *H*. *nana* eggs.

### 2.7. Data Analysis

The data were initially entered into Microsoft Excel, cleaned, and subsequently transferred to SPSS Version 25 (IBM Corp., Armonk, New York, United States) for statistical analysis. Descriptive analysis was performed to summarize the characteristics of the study participants, and categorical variables were presented using frequencies and percentages. Bivariate logistic regression analysis was conducted to assess the association between each independent variable and *H. nana* infection, and crude odds ratios (CORs) with 95% confidence intervals (CIs) were calculated. Variables with a *p* value < 0.25 in the bivariate analysis, namely age of participant, gender, location of residence, mother′s education level, father′s education level, family size, source of drinking water, playing on unclean ground, fingernail‐biting habits, type of toilet, handwashing practice, washing vegetables and fruits before consumption, and eating unwashed fruits, were entered into the multivariate logistic regression model using the enter method. Adjusted odds ratios (AORs) with 95% CIs were computed, and variables with a *p* value < 0.05 in the multivariate analysis were considered statistically significant.

### 2.8. Ethical Considerations

Ethical approval for the study was obtained from SIMAD University before commencing the research. The research proposal was reviewed and approved by the SIMAD University Research and Ethics Committee (Approval Reference: 2025/SU‐IRB/FMHS/P0030). Both written and verbal consent were obtained from the parents of all enrolled children prior to their participation in the study.

## 3. Results

### 3.1. Sociodemographic Characteristics

A total of 384 respondents comprising 206 males (53.6%) and 178 females (46.4) participated in the study. Most (161/384) of the children, with 41.9% under 5years old, 30.2% between 6 and 10 years old, and 27.9% older than 11 years. Among 384 children, 200 (52.1%) were the residents of Jilyaal camp, and 184 (47.9%) were the residents of Iga‐Daba Geey camp. Educational levels are notably low among respondents, with 77.6% being illiterate, 21.4% having primary education, and only 1% reaching secondary school. The educational attainment of parents is similarly low, with 92.4% of mothers and 88.3% of fathers being illiterate. A large majority of families (83.9%) have more than five members. The results of sociodemographic data were presented in (Table 1).

**Table 1:** Sociodemographic characteristics of children in IDPs camp at Deynile district, Mogadishu, Somalia during April 2024.

Variables	Frequency	Percent (%)
**Sex**		
Male	206	53.6
Female	178	46.4
**Age**		
<5 years	161	41.9
6–10 years	116	30.2
> 11 years	107	27.9
**Location of residence**		
Iga‐Daba Geey	184	47.9
Jilyaal	200	52.1
**Level of education of respondent**		
Illiterate	298	77.6
Primary school	82	21.4
Secondary school	4	1.0
**Mother education level**		
Illiterate	355	92.4
Primary school	28	7.3
High school and above	1	0.3
**Father education level**		
Illiterate	339	88.3
Primary school	42	10.9
High school and above	3	0.8
**Family size**		
< 5 members	62	16.1
> 5 members	322	83.9

### 3.2. Clinical Symptoms Among Respondents

Table 2 showed the prevalence of clinical symptoms among children. Diarrhea and abdominal pain are the most common, reported by 54.2% and 52.9% of respondents, respectively. Vomiting is less common, affecting 10.9% of respondents. Nausea and anorexia are rare, reported by 3.9% and 2.3% of respondents, respectively. Additionally, 4.7% of respondents experienced other symptoms.

**Table 2:** Clinical symptoms among children in IDPs camp in Deynile district, Mogadishu, Somalia during April 2024.

Variable	Category	Frequency	Percent (%)
Diarrhea	Yes	208	54.2
	No	176	45.8
Abdominal pain	Yes	203	52.9
	No	181	47.1
Vomiting	Yes	42	10.9
	No	342	89.1
Nausea	Yes	15	3.9
	No	369	96.1
Anorexia	Yes	9	2.3
	No	375	97.7
Others	Yes	18	4.7
	No	366	95.3

### 3.3. Factors Associated With *H. nana* Infections

Table 3 presents the findings from the bivariate and multivariate logistic regression analyses of factors associated with *H. nana* infection among children in the IDP camps. The study identified several significant determinants of infection. The logistic regression analysis revealed that gender, location of residence, and family size are major factors. Males are significantly more likely to be infected (AOR = 2.80; *p* = 0.003), and children from Iga‐Daba Geey have a higher likelihood of infection compared with those from Jilyaal (AOR = 4.63; *p* = 0.004). Additionally, children from larger families (> 5 members) showed a higher risk of infection (AOR = 6.96; *p* < 0.0001).

**Table 3:** Bivariate and multivariate logistic regression analysis of risk factors associated with *H. nana* infection among children in IDPs camp in Deynile district, Mogadishu, Somalia during April 2024.

Variables	N (%)	*H. nana* results		95% COR	p value	95% AOR	p value
		Positive N (%)	Negative N (%)				
**Age of participant**							
< 5 years	161 (41.9%)	35 (33.7%)	126 (45.0%)	1			
6–10 years	116 (30.2%)	38 (36.5%)	78 (27.9%)	1.46 (0.83–2.57)	0.180		
> 11 years	107 (27.9%)	31(29.8%)	76 (27.1%)	0.83 (0.47–1.48)	0.541		
**Gender of respondents**							
Male	206 (53.6%)	72 (69.2%)	134 (47.9%)	2.45 (1.50–3.95)	0.00023	2.80 (1.42–5.53)	0.003 ^∗^
Female	178 (46.4%)	32 (30.8%)	146 (46.4%)	1		1	
**Location of residence**							
Iga‐Daba Geey	184 (47.9%)	85 (81.7%)	99 (35.4%)	0.12 (0.07–0.21)	1.0925	4.63 (1.64–13.06)	0.004 ^∗^
Jilyaal	200 (52.1%)	19 (18.3%)	181 (64.6%)	1		1	
**Level of education of respondent**							
Illiterate	298 (77.6%)	79 (76.0%)	219 (78.2%)	1.136 (0.668–1.93)	0.638085		
Primary school	86 (22.4%)	25 (24.0%)	61 (21.8%)	1			
**Mother education level**							
Illiterate	355 (92.4%)	101 (97.1%)	254 (90.7%)	3.446 (1.02–11.6)	0.04632	2.76 (0.65–11.66)	0.167
Primary school	29 (7.6%)	3 (2.9%)	26 (9.3%)	1		1	
**Father education level**							
Illiterate	339 (88.3%)	99 (95.2%)	240 (85.7%)	0.30 (0.11–0.79)	0.01465	2.32 (0.73–7.34)	0.150
Primary school	45 (11.7%)	5 (4.8%)	40 (13.2%)	1			
**Family size**							
< 5 members	62 (16.1%)	5 (4.8%)	57 (20.4%)	1		1	
> 5 members	322 (83.9%)	99 (95.2%)	223 (79.6%)	0.198 (0.07–0.50)	0.00076	6.96 (2.38–20.37)	< 0.0001 ^∗^
**Source of drinking water**							
Tap water	61 (15.9%)	29 (27.9%)	32 (11.4%)	1		1	
Well water	323 (84.1%)	75 (72.1%)	248 (88.6%)	2.99 (1.70–5.27)	0.00014	0.46 (0.19–1.13)	0.093
**Contact with soil**							
Yes	368 (95.8%)	101 (97.1%)	267 (95.4%)	0.61 (0.17–2.18)	0.448		
No	16 (4.2%)	3 (2.9%)	13 (4.6%)	1			
**Playing in ground**							
Not clean	346 (90.1%)	100 (96.2%)	246 (87.9%)	0.28 (0.10–0.83)	0.02209	3.92 (1.14–13.48)	0.030
Clean	38 (9.9%)	4 (3.8%)	34 (12.1%)	1		1	
**Fingernail-biting habits**							
Yes	241 (62.8%)	28 (26.9%)	213 (76.1%)	1		1	
No	143 (37.2%)	76 (73.1%)	67 (23.9%)	8.62 (5.16–14.41)	1.7989	3.25 (1.52–6.93)	0.002 ^∗^
**Type of toilet**							
Open defecation	96 (25.0%)	31 (29.8%)	65 (23.2%)	0.71 (0.43–1.17)	0.185948	0.39 (0.15–1.02)	0.056
Public	288 (75.0%)	73 (70.2%)	215 (76.8%)	1			
**Washing hands after toilet and before meals**							
Every time	74 (19.3%)	13 (12.5%)	61 (21.8%)	1		1	
Sometimes	177 (46.1%)	63 (60.6%)	114 (40.7%)	1.25 (0.6–2.95)	0.54	0.37 (0.13–1.05)	0.063
Never	133 (34.6%)	28 (26.9%)	105 (37.5%)	0.48 (0.28–0.81)	0.006	0.90 (0.35–2.27)	0.824
**Washing vegetables and fruits before consumption**							
Yes	200 (52.1%)	17 (16.3%)	183 (65.4%)	1		1	
No	184 (47.9%)	87 (83.7%)	97 (34.6%)	9.65(5.43–17.16)	< 0.0001	8.16 (4.17–15.08)	< 0.0001 ^∗^
**Eating unwashed fruits**							
Yes	126 (32.8%)	43 (41.3%)	83 (29.6%)	0.59 (0.37–0.95)	0.030766	1.0 (0.51–1.97)	0.981
No	258 (67.2%)	61 (58.7%)	197 (70.4%)	1		1	

*Note:* 1, referent.

Abbreviations: AOR, adjusted odds ratio; CI, confidence interval; COR, crude odds ratio.

^∗^Significant association.

Other significant factors include hygiene practices and environmental conditions. Playing on unclean grounds was found associated with a higher risk of infection (AOR = 3.92; *p* = 0.030), and nail‐biting habits were strongly linked to *H. nana* infection (AOR = 3.25; *p* = 0.002). The consumption of unwashed vegetables and fruits significantly increased the risk of infection (AOR 8.16; *p* = 0.0001).

The study also highlighted that although some variables showed associations in the bivariate analysis, they were not significant in the multivariate analysis, including the source of drinking water and the education levels of parents.

## 4. Discussion

This is the first study to investigate the prevalence of *H. nana* and its associated risk factors among children living in displacement communities in the Benadir Region of Somalia. The results reveal a relatively high prevalence rate of 27.08%, representing the highest documented rate in the Somali Region to date. In comparison, previous studies conducted among children in various regions of Somalia reported significantly lower prevalence rates, ranging between 0.3% and 18.5% [8, 23–25]. The variation in these findings may be attributed to several factors, including differences in geographical location, characteristics of the target populations, climatic differences, levels of environmental hygiene, history of deworming or public health interventions, socioeconomic conditions, and variations in host immunity or susceptibility to infection.

Our study result was in line with several studies conducted in Mexico [26], Ethiopia [27], as well as in urban areas of Zimbabwe [28]. However, the present study was lower to studies conducted among preschool children of displacement communities in Khartoum state, Sudan [9] and Afghan refugee populations in Mianwali district, Pakistan [4]. This suggests that populations displaced due to conflict and residing in IDP camps may face even greater risk due to heightened vulnerability and severely limited access to basic health services and sanitation.

The findings revealed several factors significantly associated with *H. nana* infection. Among the sociodemographic characteristics, gender, place of residence, and family size were found to be statistically significant.

In terms of gender, male children were significantly more likely to be infected with *H. nana* compared with females (AOR = 2.80; 95% CI: 1.42–5.53; *p* = 0.003). This result is consistent with findings from a study conducted among preschool children in displaced communities in Khartoum, Sudan, which also reported a statistically significant higher prevalence among males than females [9]. One possible reason for this could be that boys tend to spend more time playing outside, where they may encounter contaminated soil or environments, increasing their risk of infection through behaviors like touching their mouths or not washing their hands properly afterward.

Interestingly, this study found a significant association between place of residence and *H. nana* infection. Children living in Iga‐Daba Geey were significantly more likely to be infected compared with those residing in Jilyaal (AOR = 4.63; 95% CI: 1.64–13.06; *p* = 0.004). This finding is consistent with a study conducted in rural Yemen [13], which also reported that place of residence was significantly associated with the risk of *H. nana* infection. This may be due to several contributing factors. Iga‐Daba Geey is more densely populated and geographically expansive than Jilyaal, and it is also one of the older IDP settlements in the area. Over time, such camps often experience greater strain on their infrastructure, including sanitation and access to clean water. These conditions increase the risk of parasite transmission, particularly among children who frequently interact with contaminated environments. Therefore, this highlights the urgent need for targeted environmental health interventions and infrastructure improvements in high‐risk IDP settlements such as Iga‐Daba Geey.

In terms of family size, the present study found that the family size was associated with *H. nana* infection (AOR = 6.96, 95% CI: 2.38–20.37; *p* = 0.0001). This finding was aligned with similar study conducted in Egypt [6]. A possible explanation is that larger families often experience overcrowding, which can lead to increased person‐to‐person transmission through shared living spaces, limited access to sanitation facilities, and challenges in maintaining personal hygiene.

Regarding environmental factors, this study found that children who played in unclean grounds were significantly more likely to be infected with *H. nana* compared with those who played in clean environments (AOR = 3.92; 95% CI: 1.14–13.48; *p* = 0.030). This finding is consistent with a study conducted in the Cusco Region of Peru, where similar associations were reported between soil exposure and *H. nana* infection [5]. This suggests that contaminated play areas are a major risk factor for transmission, likely due to the presence of parasite eggs in soil and fecal matter and children often play barefoot, touch contaminated surfaces, or put their hands in their mouths, and these behaviors may increase the risk of ingesting infective eggs.

In the present study, fingernail‐biting habits were found to be significantly associated with *H. nana* infection. Children who had a habit of biting their fingernails were 3.25 times more likely to test positive for *H. nana* compared with those without this habit (AOR = 3.25; 95% CI: 1.52–6.93; *p* = 0.002). This finding is consistent with a study conducted in Nepal [29–31], which also reported that nail‐biting behavior significantly increased the risk of *H. nana* infection. A possible explanation is that fingernail‐biting behavior facilitates the direct ingestion of parasite eggs, particularly in children who are frequently exposed to contaminated environments and have poor hand hygiene.

The consumption of unwashed vegetables and fruits significantly raises the risk of *H. nana* infection. Children who had a habit of consumption of unwashed vegetables and fruits were 8.16 times more likely to test positive for *H. nana* compared with those without this habit (AOR = 8.16; 95% CI: 4.17–15.08; *p* = 0.0001). This finding is consistent with a study conducted in the Sohag, Egypt [6], which also reported that consumption of unwashed vegetables and fruits behavior significantly increased the risk of *H. nana* infection due to the potential for oral‐fecal contamination.

The study also highlighted that although some variables showed associations in the bivariate analysis, they were not significant in the multivariate analysis, including the source of drinking water and the education levels of parents. This suggests that although these factors may contribute to infection risk, they are not considered as influential as the identified significant factors when considering multiple variables simultaneously. Addressing the major risk factors through improved sanitation, hygiene practices, and education could substantially reduce the prevalence of *H. nana* infections among children in these displacement communities. These findings highlight the need for targeted public health interventions to control *H*. *nana* infection among children in IDP camps. Strengthening WASH services, promoting hygiene and safe food‐handling practices, and improving clean play environments are essential priorities for NGOs, UN agencies, and other humanitarian partners. In addition, the Somali Ministry of Health should work with these stakeholders to integrate routine screening, periodic deworming, and improved diagnostic and surveillance capacity into primary healthcare services in IDP camps to reduce the burden of infection in this vulnerable population.

## 5. Limitations

Several limitations should be considered when interpreting these findings. First, participants were recruited from selected IDP camps in Somalia using convenience sampling, which may introduce selection bias and limits generalizability to other IDP camps, host communities, or nondisplaced populations. Second, diagnosis was based on a single stool specimen examined by saline wet mount microscopy. Although more sensitive methods (e.g., Kato–Katz or concentration techniques) are recommended, Kato–Katz was not available in the study setting and concentration techniques were not feasible due to local laboratory and regulatory constraints; therefore, prevalence may be underestimated, particularly for low‐intensity infections. Third, because quantitative diagnostic methods were unavailable, infection intensity (eggs per gram of stool) could not be assessed, limiting evaluation of infection burden and related clinical implications. Despite these constraints, the study provides important baseline data from a resource‐limited humanitarian context in Somalia.

## 6. Conclusions

This study revealed a relatively high prevalence of *H. nana* infection among children living in IDP settlements in the Benadir Region of Somalia, representing the highest rate reported in the country to date. Compared with earlier findings from other regions, this elevated prevalence highlights an important public health concern in displacement settings. Several factors were significantly associated with *H. nana* infection, including male sex, place of residence, larger family size, playing in unclean environments, fingernail‐biting habits, and the consumption of unwashed vegetables and fruits. Targeted public health interventions are therefore needed, including hygiene education on handwashing and safe food handling, discouraging nail‐biting, and improving sanitation, access to clean water, and safe play areas in IDP settlements. There is an urgent need to integrate routine deworming and health screenings into community health programs. Such integration is crucial to reduce the burden of *H. nana* infection among vulnerable children in IDP camps.

## Author Contributions

A.O.S. and A.M.S.M. designed the study. M.A.M., N.A.I., and A.A.A. performed the study including sample collection and laboratory analysis. J.H.M. and M.H.A. analyzed the data. M.A.M., B.G., and A.E. wrote the first draft and revised the paper.

## Funding

No funding was received for this manuscript.

## Disclosure

All authors read and approved the final draft of the manuscript.

## Conflicts of Interest

The authors declare no conflicts of interest.

## Data Availability

All data available on request from the authors (qariskatuur@simad.edu.so).
